# Supplementary material for: Imputation by feature importance (IBFI): A methodology to envelop machine learning method for imputing missing patterns in time series data
Source: PLoS One. 2022 Jan 13;17(1):e0262131. doi: 10.1371/journal.pone.0262131 (PMC8758196; doi:10.1371/journal.pone.0262131)
Supplement: S1 Table — Summary of the results of the simulations at missing completely at random (MCAR) with the missingness percentage of a) 10%, b) 20%, and c) 30. (DOCX) [file pone.0262131.s001.docx]

S1 Table: Summary of the results of the simulations at missing completely at random (MCAR) with the missingness percentage of a) 10%, b) 20%, and c) 30%

a)

| Imputation Methods | Performance  Measures | RN | TH | TC | RH | PR | Average |
| --- | --- | --- | --- | --- | --- | --- | --- |
| Proposed Methodology | RMSE | 322.2269 | 42.0663 | 0.5335 | 0.7406 | 0.4795 | 73.2094 |
|  | RMSLE | 0.0155 | 0.0169 | 0.0309 | 0.0098 | 0.0005 | 0.0147 |
|  | MAPE | 0.0024 | 0.0023 | 0.0042 | 0.0011 | 0.0001 | 0.002 |
|  | PB | -0.0002 | -0.0003 | -0.0007 | -0.0001 | 0 | -0.0003 |
|  | MSE | 103830.2 | 1769.575 | 0.2847 | 0.5484 | 0.2299 | 21120.16 |
| Mean Imputation | RMSE | 404.2185 | 59.9236 | 1.4583 | 1.8139 | 0.752 | 93.6333 |
|  | RMSLE | 0.0196 | 0.024 | 0.0716 | 0.025 | 0.0008 | 0.0282 |
|  | MAPE | 0.0031 | 0.0034 | 0.0131 | 0.003 | 0.0001 | 0.0045 |
|  | PB | -0.0003 | -0.0006 | -0.0058 | -0.0005 | 0 | -0.0015 |
|  | MSE | 163392.6 | 3590.843 | 2.1266 | 3.2903 | 0.5656 | 33397.89 |
| Median Imputation | RMSE | 405.4279 | 60.0236 | 1.4586 | 1.8474 | 0.7588 | 93.9033 |
|  | RMSLE | 0.0197 | 0.0239 | 0.0723 | 0.0258 | 0.0008 | 0.0285 |
|  | MAPE | 0.0031 | 0.0034 | 0.0134 | 0.003 | 0.0001 | 0.0046 |
|  | PB | -0.0007 | -0.0003 | -0.0067 | -0.0013 | 0 | -0.0018 |
|  | MSE | 164371.8 | 3602.827 | 2.1275 | 3.4129 | 0.5757 | 33596.15 |
| Mode Imputation | RMSE | 471.8653 | 62.0839 | 2.6178 | 2.4372 | 0.9154 | 107.9839 |
|  | RMSLE | 0.0223 | 0.0244 | 0.1361 | 0.0328 | 0.001 | 0.0433 |
|  | MAPE | 0.0037 | 0.0034 | 0.0156 | 0.004 | 0.0001 | 0.0054 |
|  | PB | 0.0019 | 0.0006 | 0.0142 | -0.0037 | 0.0001 | 0.0026 |
|  | MSE | 222656.9 | 3854.405 | 6.8527 | 5.9397 | 0.838 | 45304.98 |
| PMM Imputation | RMSE | 507.4033 | 61.933 | 0.7659 | 1.0777 | 0.7271 | 114.3814 |
|  | RMSLE | 0.0244 | 0.0248 | 0.0443 | 0.0145 | 0.0008 | 0.0218 |
|  | MAPE | 0.0039 | 0.0034 | 0.0061 | 0.0016 | 0.0001 | 0.003 |
|  | PB | -0.0001 | -0.0004 | -0.0011 | 0 | 0 | -0.0003 |
|  | MSE | 257458.1 | 3835.691 | 0.5867 | 1.1615 | 0.5287 | 52259.22 |
| Hotdeck Imputation | RMSE | 600.8383 | 85.2137 | 2.0955 | 2.4017 | 1.0635 | 138.3225 |
|  | RMSLE | 0.0288 | 0.034 | 0.103 | 0.0334 | 0.0011 | 0.0401 |
|  | MAPE | 0.0046 | 0.0048 | 0.0167 | 0.0037 | 0.0001 | 0.006 |
|  | PB | -0.0003 | -0.0002 | -0.0054 | -0.0007 | 0 | -0.0013 |
|  | MSE | 361006.6 | 7261.376 | 4.3912 | 5.7683 | 1.1309 | 73655.86 |

**RN:** Radon Concentration; **TH:** Thoron; **TC:** Temperature in degree Celsius; **RH:** Relative Humidity; **PR:** Pressure; **PMM:** Predictive mean matching

b)

| Imputation Methods | Performance  Measures | RN | TH | TC | RH | PR | Average |
| --- | --- | --- | --- | --- | --- | --- | --- |
| Proposed Methodology | RMSE | 492.4559 | 59.7265 | 0.7072 | 1.0956 | 0.723 | 110.9416 |
|  | RMSLE | 0.0237 | 0.0241 | 0.0421 | 0.0149 | 0.0008 | 0.0211 |
|  | MAPE | 0.0053 | 0.0048 | 0.0083 | 0.0024 | 0.0001 | 0.0042 |
|  | PB | -0.0004 | -0.0005 | -0.0018 | -0.0001 | 0 | -0.0006 |
|  | MSE | 242512.8 | 3567.251 | 0.5001 | 1.2004 | 0.5228 | 49216.46 |
| Mean Imputation | RMSE | 615.027 | 87.451 | 2.0996 | 2.9474 | 1.1054 | 141.7261 |
|  | RMSLE | 0.0295 | 0.035 | 0.1053 | 0.0425 | 0.0012 | 0.0427 |
|  | MAPE | 0.0069 | 0.0072 | 0.0278 | 0.0074 | 0.0002 | 0.0099 |
|  | PB | -0.0005 | -0.0011 | -0.0138 | -0.0018 | 0 | -0.0034 |
|  | MSE | 378258.2 | 7647.673 | 4.4085 | 8.6874 | 1.222 | 77184.03 |
| Median Imputation | RMSE | 615.2225 | 87.7807 | 2.1064 | 3.0103 | 1.1209 | 141.8482 |
|  | RMSLE | 0.0297 | 0.035 | 0.1065 | 0.0439 | 0.0012 | 0.0433 |
|  | MAPE | 0.0069 | 0.0071 | 0.0284 | 0.0075 | 0.0002 | 0.01 |
|  | PB | -0.0013 | -0.0005 | -0.0156 | -0.0036 | 0 | -0.0042 |
|  | MSE | 378498.7 | 7705.454 | 4.4367 | 9.0621 | 1.2564 | 77243.78 |
| Mode Imputation | RMSE | 643.6853 | 140.6457 | 3.5549 | 3.0103 | 1.3226 | 158.4438 |
|  | RMSLE | 0.0305 | 0.0575 | 0.1816 | 0.0439 | 0.0014 | 0.063 |
|  | MAPE | 0.0072 | 0.0107 | 0.0301 | 0.0075 | 0.0003 | 0.0112 |
|  | PB | 0.0017 | 0.0098 | 0.0261 | -0.0018 | 0.0002 | 0.0072 |
|  | MSE | 414330.8 | 19781.2 | 12.6374 | 8.6874 | 1.7492 | 86827.02 |
| PMM Imputation | RMSE | 738.0973 | 139.1167 | 1.1279 | 1.5245 | 1.0252 | 176.1783 |
|  | RMSLE | 0.0351 | 0.0377 | 0.0652 | 0.0205 | 0.0011 | 0.0319 |
|  | MAPE | 0.0081 | 0.0071 | 0.0128 | 0.0033 | 0.0002 | 0.0063 |
|  | PB | -0.0009 | -0.0011 | -0.0024 | -0.0001 | 0 | -0.0009 |
|  | MSE | 544787.6 | 19353.47 | 1.2721 | 2.3241 | 1.0511 | 112829.1 |
| Hotdeck Imputation | RMSE | 874.8935 | 121.0264 | 2.9213 | 4.0907 | 1.5881 | 200.904 |
|  | RMSLE | 0.0423 | 0.0481 | 0.1436 | 0.0586 | 0.0017 | 0.0589 |
|  | MAPE | 0.0095 | 0.0098 | 0.0347 | 0.0096 | 0.0003 | 0.0128 |
|  | PB | -0.0002 | -0.0014 | -0.0134 | -0.0013 | 0 | -0.0032 |
|  | MSE | 765438.6 | 14647.39 | 8.5342 | 16.7341 | 2.522 | 156022.7 |

**RN:** Radon Concentration; **TH:** Thoron; **TC:** Temperature in degree Celsius; **RH:** Relative Humidity; **PR:** Pressure; **PMM:** Predictive mean matching

c)

| Imputation Methods | Performance  Measures | RN | TH | TC | RH | PMBR | Average |
| --- | --- | --- | --- | --- | --- | --- | --- |
| Proposed Methodology | RMSE | 597.3742 | 90.3982 | 0.8968 | 1.3313 | 0.8596 | 138.172 |
|  | RMSLE | 0.0289 | 0.0312 | 0.0512 | 0.0181 | 0.0009 | 0.0261 |
|  | MAPE | 0.0079 | 0.0073 | 0.012 | 0.0034 | 0.0002 | 0.0062 |
|  | PB | -0.001 | -0.0007 | -0.0023 | -0.0003 | 0 | -0.0009 |
|  | MSE | 356855.9 | 8171.827 | 0.8043 | 1.7723 | 0.739 | 73006.21 |
| Mean Imputation | RMSE | 769.1196 | 121.7373 | 2.543 | 3.2915 | 1.3731 | 179.6129 |
|  | RMSLE | 0.0374 | 0.045 | 0.1244 | 0.0464 | 0.0015 | 0.0509 |
|  | MAPE | 0.0108 | 0.0114 | 0.0388 | 0.0097 | 0.0003 | 0.0142 |
|  | PB | -0.0019 | -0.0014 | -0.0171 | -0.0023 | 0 | -0.0046 |
|  | MSE | 591545 | 14819.97 | 6.467 | 10.8338 | 1.8853 | 121276.8 |
| Median Imputation | RMSE | 775.1135 | 122.3052 | 2.542 | 3.3876 | 1.3936 | 180.9484 |
|  | RMSLE | 0.0378 | 0.045 | 0.1256 | 0.0483 | 0.0015 | 0.0516 |
|  | MAPE | 0.0108 | 0.0113 | 0.0396 | 0.0098 | 0.0003 | 0.0144 |
|  | PB | -0.0031 | -0.0004 | -0.0198 | -0.0049 | -0.0001 | -0.0057 |
|  | MSE | 600801 | 14958.56 | 6.4618 | 11.4756 | 1.9421 | 123155.9 |
| Mode Imputation | RMSE | 831.9415 | 185.9884 | 4.5144 | 4.4647 | 1.6247 | 205.7067 |
|  | RMSLE | 0.0396 | 0.0733 | 0.2334 | 0.0609 | 0.0017 | 0.0818 |
|  | MAPE | 0.0115 | 0.0166 | 0.0456 | 0.0129 | 0.0004 | 0.0174 |
|  | PB | 0.004 | 0.0154 | 0.0411 | -0.0122 | 0.0003 | 0.0097 |
|  | MSE | 692126.7 | 34591.68 | 20.3795 | 19.9334 | 2.6398 | 145352.3 |
| PMM Imputation | RMSE | 926.6903 | 115.709 | 1.332 | 1.9846 | 1.2782 | 209.3988 |
|  | RMSLE | 0.0444 | 0.0429 | 0.0766 | 0.0275 | 0.0014 | 0.0386 |
|  | MAPE | 0.0126 | 0.0104 | 0.018 | 0.0053 | 0.0003 | 0.0093 |
|  | PB | -0.0011 | -0.0007 | -0.0033 | -0.0005 | 0 | -0.0011 |
|  | MSE | 858754.9 | 13388.58 | 1.7742 | 3.9385 | 1.6338 | 174430.2 |
| Hotdeck Imputation | RMSE | 1077.115 | 168.9251 | 3.5351 | 4.5932 | 1.9313 | 251.2199 |
|  | RMSLE | 0.0521 | 0.0632 | 0.1714 | 0.0646 | 0.0021 | 0.0707 |
|  | MAPE | 0.0147 | 0.0158 | 0.0492 | 0.0125 | 0.0005 | 0.0185 |
|  | PB | -0.0013 | -0.0018 | -0.0168 | -0.0021 | 0 | -0.0044 |
|  | MSE | 1160176 | 28535.7 | 12.4972 | 21.0977 | 3.73 | 237749.9 |

**RN:** Radon Concentration; **TH:** Thoron; **TC:** Temperature in degree Celsius; **RH:** Relative Humidity; **PR:** Pressure; **PMM:** Predictive mean matching
