# Supplementary material for: Imputation by feature importance (IBFI): A methodology to envelop machine learning method for imputing missing patterns in time series data
Source: PLoS One. 2022 Jan 13;17(1):e0262131. doi: 10.1371/journal.pone.0262131 (PMC8758196; doi:10.1371/journal.pone.0262131)
Supplement: S2 Table — Summary of the results of the simulations at missing not at random (MNAR) with the missingness percentage of a) 10%, b) 20%, and c) 30. (DOCX) [file pone.0262131.s002.docx]

S2 Table: Summary of the results of the simulations at missing not at random (MNAR) with the missingness percentage of a) 10%, b) 20%, and c) 30%

a)

| Imputation Methods | Performance  Measures | RN | TH | TC | RH | PMBR | Average |
| --- | --- | --- | --- | --- | --- | --- | --- |
| Proposed Methodology | RMSE | 388.6556 | 58.8726 | 0.488 | 0.7459 | 0.5489 | 89.8622 |
|  | RMSLE | 0.0172 | 0.0204 | 0.0224 | 0.0092 | 0.0006 | 0.014 |
|  | MAPE | 0.0027 | 0.0027 | 0.0027 | 0.001 | 0.0001 | 0.0018 |
|  | PB | 0.0016 | 0.0014 | 0.0009 | 0.0003 | 0 | 0.0009 |
|  | MSE | 151053.2 | 3465.98 | 0.2381 | 0.5564 | 0.3013 | 30904.05 |
| Mean Imputation | RMSE | 492.3819 | 84.74 | 1.534 | 1.7022 | 0.9069 | 116.253 |
|  | RMSLE | 0.0218 | 0.0302 | 0.0586 | 0.0205 | 0.001 | 0.0264 |
|  | MAPE | 0.0036 | 0.0043 | 0.0088 | 0.0025 | 0.0001 | 0.0038 |
|  | PB | 0.0025 | 0.0025 | 0.0031 | 0.0016 | 0.0001 | 0.002 |
|  | MSE | 242440 | 7180.875 | 2.3533 | 2.8973 | 0.8226 | 49925.38 |
| Median Imputation | RMSE | 467.3025 | 87.5081 | 1.5143 | 1.4404 | 0.8446 | 111.722 |
|  | RMSLE | 0.0207 | 0.0312 | 0.0579 | 0.0175 | 0.0009 | 0.0256 |
|  | MAPE | 0.0034 | 0.0044 | 0.0087 | 0.0021 | 0.0001 | 0.0037 |
|  | PB | 0.0021 | 0.0028 | 0.0029 | 0.0008 | 0.0001 | 0.0017 |
|  | MSE | 218371.7 | 7657.669 | 2.2932 | 2.0747 | 0.7134 | 45206.88 |
| Mode Imputation | RMSE | 561.3097 | 143.6531 | 2.8103 | 1.3275 | 1.2617 | 142.0724 |
|  | RMSLE | 0.025 | 0.0564 | 0.1278 | 0.0168 | 0.0014 | 0.0455 |
|  | MAPE | 0.0042 | 0.0078 | 0.0152 | 0.0017 | 0.0002 | 0.0058 |
|  | PB | 0.0034 | 0.0077 | 0.0147 | -0.0008 | 0.0002 | 0.005 |
|  | MSE | 315068.5 | 20636.21 | 7.8978 | 1.7622 | 1.5919 | 67143.2 |
| PMM Imputation | RMSE | 555.4801 | 72.2747 | 0.7118 | 1.0866 | 0.7654 | 126.0637 |
|  | RMSLE | 0.0256 | 0.0264 | 0.0355 | 0.0134 | 0.0008 | 0.0203 |
|  | MAPE | 0.004 | 0.0035 | 0.0041 | 0.0014 | 0.0001 | 0.0026 |
|  | PB | 0.0018 | 0.0013 | 0.0007 | 0.0003 | 0 | 0.0008 |
|  | MSE | 308558.1 | 5223.628 | 0.5067 | 1.1808 | 0.5858 | 62756.8 |
| Hotdeck Imputation | RMSE | 668.6311 | 105.4504 | 2.0892 | 2.6733 | 1.2316 | 156.0151 |
|  | RMSLE | 0.0313 | 0.0401 | 0.0945 | 0.0361 | 0.0013 | 0.0406 |
|  | MAPE | 0.0048 | 0.0053 | 0.0117 | 0.0034 | 0.0002 | 0.0051 |
|  | PB | 0.0024 | 0.0025 | 0.004 | 0.0016 | 0.0001 | 0.0021 |
|  | MSE | 447067.5 | 11119.78 | 4.3646 | 7.1467 | 1.5168 | 91640.07 |

**RN:** Radon Concentration; **TH:** Thoron; **TC:** Temperature in degree Celsius; **RH:** Relative Humidity; **PR:** Pressure; **PMM:** Predictive mean matching

b)

| Imputation Methods | Performance  Measures | RN | TH | TC | RH | PMBR | Average |
| --- | --- | --- | --- | --- | --- | --- | --- |
| Proposed Methodology | RMSE | 539.358 | 99.8049 | 0.6717 | 0.9838 | 0.7001 | 128.3037 |
|  | RMSLE | 0.0242 | 0.0282 | 0.0312 | 0.012 | 0.0008 | 0.0193 |
|  | MAPE | 0.0052 | 0.0046 | 0.0054 | 0.0018 | 0.0001 | 0.0034 |
|  | PB | 0.0031 | 0.0021 | 0.0012 | 0.0007 | 0.0001 | 0.0014 |
|  | MSE | 290907 | 9961.027 | 0.4512 | 0.9678 | 0.4901 | 60174 |
| Mean Imputation | RMSE | 657.8613 | 126.876 | 2.0615 | 2.3427 | 1.1275 | 158.0538 |
|  | RMSLE | 0.0294 | 0.0406 | 0.0819 | 0.0281 | 0.0012 | 0.0362 |
|  | MAPE | 0.0067 | 0.0075 | 0.0171 | 0.0047 | 0.0002 | 0.0073 |
|  | PB | 0.0042 | 0.0042 | 0.0046 | 0.0034 | 0.0001 | 0.0033 |
|  | MSE | 432781.5 | 16097.53 | 4.2499 | 5.4881 | 1.2712 | 89778.01 |
| Median Imputation | RMSE | 626.3946 | 129.6234 | 2.02 | 1.916 | 1.0482 | 152.2004 |
|  | RMSLE | 0.028 | 0.0417 | 0.0806 | 0.0232 | 0.0011 | 0.0349 |
|  | MAPE | 0.0063 | 0.0077 | 0.017 | 0.0037 | 0.0002 | 0.007 |
|  | PB | 0.0035 | 0.0047 | 0.0038 | 0.0018 | 0.0001 | 0.0028 |
|  | MSE | 392370.1 | 16802.21 | 4.0804 | 3.671 | 1.0988 | 81836.24 |
| Mode Imputation | RMSE | 819.2895 | 202.058 | 3.7674 | 1.6966 | 1.5913 | 205.6806 |
|  | RMSLE | 0.037 | 0.0766 | 0.1733 | 0.0214 | 0.0017 | 0.062 |
|  | MAPE | 0.0086 | 0.0142 | 0.0291 | 0.003 | 0.0003 | 0.0111 |
|  | PB | 0.0073 | 0.014 | 0.0271 | -0.0014 | 0.0003 | 0.0095 |
|  | MSE | 671235.3 | 40827.42 | 14.1936 | 2.8784 | 2.5322 | 142416.5 |
| PMM Imputation | RMSE | 762.6739 | 120.3583 | 0.9981 | 1.4242 | 1.0435 | 177.2996 |
|  | RMSLE | 0.0353 | 0.0388 | 0.048 | 0.018 | 0.0011 | 0.0282 |
|  | MAPE | 0.0077 | 0.0067 | 0.0082 | 0.0026 | 0.0002 | 0.0051 |
|  | PB | 0.0032 | 0.0021 | 0.001 | 0.0008 | 0.0001 | 0.0014 |
|  | MSE | 581671.5 | 14486.12 | 0.9962 | 2.0283 | 1.089 | 119232.3 |
| Hotdeck Imputation | RMSE | 885.3978 | 148.5672 | 2.7746 | 3.5318 | 1.5244 | 208.3592 |
|  | RMSLE | 0.0417 | 0.0522 | 0.1276 | 0.0481 | 0.0016 | 0.0542 |
|  | MAPE | 0.0088 | 0.0094 | 0.0224 | 0.0064 | 0.0003 | 0.0095 |
|  | PB | 0.0045 | 0.0042 | 0.004 | 0.0034 | 0.0001 | 0.0032 |
|  | MSE | 783929.2 | 22072.22 | 7.6986 | 12.4735 | 2.3238 | 161204.8 |

**RN:** Radon Concentration; **TH:** Thoron; **TC:** Temperature in degree Celsius; **RH:** Relative Humidity; **PR:** Pressure; **PMM:** Predictive mean matching

c)

| Imputation Methods | Performance  Measures | RN | TH | TC | RH | PMBR | Average |
| --- | --- | --- | --- | --- | --- | --- | --- |
| Proposed Methodology | RMSE | 624.5544 | 147.8227 | 0.8739 | 1.3415 | 0.911 | 155.1007 |
|  | RMSLE | 0.0282 | 0.0358 | 0.0426 | 0.0166 | 0.001 | 0.0248 |
|  | MAPE | 0.0075 | 0.0069 | 0.0092 | 0.0032 | 0.0002 | 0.0054 |
|  | PB | 0.004 | 0.0026 | 0.0013 | 0.0009 | 0.0001 | 0.0018 |
|  | MSE | 390068.2 | 21851.55 | 0.7637 | 1.7998 | 0.83 | 82384.62 |
| Mean Imputation | RMSE | 755.1172 | 171.4926 | 2.5635 | 3.0108 | 1.3982 | 186.7165 |
|  | RMSLE | 0.034 | 0.0487 | 0.1045 | 0.037 | 0.0015 | 0.0451 |
|  | MAPE | 0.0095 | 0.0108 | 0.028 | 0.0079 | 0.0003 | 0.0113 |
|  | PB | 0.0052 | 0.0043 | 0.005 | 0.0042 | 0.0002 | 0.0038 |
|  | MSE | 570202 | 29409.7 | 6.5714 | 9.0652 | 1.9551 | 119925.9 |
| Median Imputation | RMSE | 722.3833 | 174.0099 | 2.5695 | 2.606 | 1.3085 | 180.5754 |
|  | RMSLE | 0.0325 | 0.0499 | 0.1047 | 0.0327 | 0.0014 | 0.0442 |
|  | MAPE | 0.0091 | 0.011 | 0.028 | 0.0067 | 0.0003 | 0.011 |
|  | PB | 0.0042 | 0.0051 | 0.0051 | 0.0014 | 0.0001 | 0.0032 |
|  | MSE | 521837.6 | 30279.45 | 6.6024 | 6.7912 | 1.7122 | 110426.4 |
| Mode Imputation | RMSE | 788.0298 | 209.1863 | 4.5935 | 2.6591 | 1.9416 | 201.2821 |
|  | RMSLE | 0.0355 | 0.0675 | 0.2123 | 0.0343 | 0.0021 | 0.0703 |
|  | MAPE | 0.01 | 0.0147 | 0.0446 | 0.0064 | 0.0005 | 0.0152 |
|  | PB | 0.0062 | 0.0129 | 0.0411 | -0.004 | 0.0004 | 0.0113 |
|  | MSE | 620991 | 43758.89 | 21.1 | 7.0709 | 3.7697 | 132956.4 |
| PMM Imputation | RMSE | 908.8475 | 163.7594 | 1.2669 | 1.9674 | 1.2937 | 215.427 |
|  | RMSLE | 0.0425 | 0.0457 | 0.0629 | 0.0248 | 0.0014 | 0.0354 |
|  | MAPE | 0.0112 | 0.0097 | 0.0135 | 0.0047 | 0.0003 | 0.0079 |
|  | PB | 0.0042 | 0.002 | 0.0014 | 0.0006 | 0.0001 | 0.0017 |
|  | MSE | 826003.7 | 26817.16 | 1.605 | 3.8708 | 1.6736 | 170565.6 |
| Hotdeck Imputation | RMSE | 1057.583 | 205.3924 | 3.489 | 4.5228 | 1.9598 | 254.5894 |
|  | RMSLE | 0.0502 | 0.0637 | 0.1616 | 0.0619 | 0.0021 | 0.0679 |
|  | MAPE | 0.0131 | 0.014 | 0.037 | 0.0107 | 0.0005 | 0.0151 |
|  | PB | 0.0052 | 0.0047 | 0.0051 | 0.0038 | 0.0002 | 0.0038 |
|  | MSE | 1118482 | 42186.04 | 12.1729 | 20.4558 | 3.8408 | 232140.9 |

**RN:** Radon Concentration; **TH:** Thoron; **TC:** Temperature in degree Celsius; **RH:** Relative Humidity; **PR:** Pressure; **PMM:** Predictive mean matching
