# Supplementary material for: Imputation by feature importance (IBFI): A methodology to envelop machine learning method for imputing missing patterns in time series data
Source: PLoS One. 2022 Jan 13;17(1):e0262131. doi: 10.1371/journal.pone.0262131 (PMC8758196; doi:10.1371/journal.pone.0262131)
Supplement: S3 Table — Summary of the results of the simulations at missing at random (MAR) with the missingness percentage of a) 10%, b) 20%, and c) 30. (DOCX) [file pone.0262131.s003.docx]

S3 Table: Summary of the results of the simulations at missing at random (MAR) with the missingness percentage of a) 10%, b) 20%, and c) 30%

a)

| Imputation Methods | Performance  Measures | RN | TH | TC | RH | PMBR | Average |
| --- | --- | --- | --- | --- | --- | --- | --- |
| Proposed Methodology | RMSE | 344.0626 | 42.3939 | 0.5412 | 0.7217 | 0.5217 | 77.6482 |
|  | RMSLE | 0.0161 | 0.0171 | 0.0332 | 0.0098 | 0.0006 | 0.0154 |
|  | MAPE | 0.0024 | 0.0024 | 0.0048 | 0.0011 | 0.0001 | 0.0021 |
|  | PB | 0 | -0.0003 | -0.0009 | -0.0001 | 0 | -0.0003 |
|  | MSE | 118379 | 1797.246 | 0.2929 | 0.5208 | 0.2721 | 24035.48 |
| Mean Imputation | RMSE | 430.0932 | 62.7497 | 1.394 | 1.8436 | 0.8332 | 99.3827 |
|  | RMSLE | 0.02 | 0.0251 | 0.0772 | 0.0272 | 0.0009 | 0.0301 |
|  | MAPE | 0.0032 | 0.0036 | 0.0149 | 0.0031 | 0.0001 | 0.005 |
|  | PB | 0.0008 | -0.0008 | -0.0109 | -0.0014 | 0 | -0.0025 |
|  | MSE | 184980.2 | 3937.523 | 1.9431 | 3.3989 | 0.6942 | 37784.74 |
| Median Imputation | RMSE | 419.863 | 62.7558 | 1.4179 | 1.9776 | 0.8764 | 97.3782 |
|  | RMSLE | 0.0196 | 0.0249 | 0.0785 | 0.0291 | 0.0009 | 0.0306 |
|  | MAPE | 0.0031 | 0.0036 | 0.0153 | 0.0033 | 0.0001 | 0.0051 |
|  | PB | 0.0004 | -0.0005 | -0.0116 | -0.0023 | -0.0001 | -0.0028 |
|  | MSE | 176284.9 | 3938.293 | 2.0105 | 3.9111 | 0.7681 | 36045.99 |
| Mode Imputation | RMSE | 545.7221 | 97.0581 | 2.1023 | 2.7406 | 0.829 | 129.6904 |
|  | RMSLE | 0.0252 | 0.0395 | 0.1149 | 0.0377 | 0.0009 | 0.0436 |
|  | MAPE | 0.0041 | 0.005 | 0.0134 | 0.0048 | 0.0001 | 0.0055 |
|  | PB | 0.0029 | 0.0046 | 0.0112 | -0.0047 | 0 | 0.0028 |
|  | MSE | 297812.6 | 9420.284 | 4.4197 | 7.5108 | 0.6872 | 61449.1 |
| PMM Imputation | RMSE | 499.2213 | 57.9832 | 0.7999 | 1.1153 | 0.7606 | 111.976 |
|  | RMSLE | 0.0233 | 0.0233 | 0.0488 | 0.0157 | 0.0008 | 0.0224 |
|  | MAPE | 0.0036 | 0.0032 | 0.007 | 0.0018 | 0.0001 | 0.0031 |
|  | PB | -0.0002 | -0.0004 | -0.0015 | -0.0003 | 0 | -0.0005 |
|  | MSE | 249221.9 | 3362.048 | 0.6398 | 1.2439 | 0.5785 | 50517.28 |
| Hotdeck Imputation | RMSE | 602.8397 | 87.7905 | 2.0206 | 2.6393 | 1.1736 | 139.2928 |
|  | RMSLE | 0.0287 | 0.0348 | 0.1025 | 0.0383 | 0.0013 | 0.0411 |
|  | MAPE | 0.0043 | 0.005 | 0.0193 | 0.0043 | 0.0002 | 0.0066 |
|  | PB | 0.0008 | -0.0012 | -0.0115 | -0.0013 | 0 | -0.0026 |
|  | MSE | 363415.8 | 7707.175 | 4.0829 | 6.9661 | 1.3774 | 74227.07 |

**RN:** Radon Concentration; **TH:** Thoron; **TC:** Temperature in degree Celsius; **RH:** Relative Humidity; **PR:** Pressure; **PMM:** Predictive mean matching

b)

| Imputation Methods | Performance  Measures | RN | TH | TC | RH | PMBR | Average |
| --- | --- | --- | --- | --- | --- | --- | --- |
| Proposed Methodology | RMSE | 471.9751 | 60.8545 | 0.7808 | 1.0695 | 0.7421 | 107.0844 |
|  | RMSLE | 0.022 | 0.0243 | 0.0472 | 0.0145 | 0.0008 | 0.0217 |
|  | MAPE | 0.005 | 0.0048 | 0.0097 | 0.0023 | 0.0001 | 0.0044 |
|  | PB | 0.0001 | -0.0003 | -0.0016 | -0.0001 | 0 | -0.0004 |
|  | MSE | 222760.5 | 3703.276 | 0.6096 | 1.1438 | 0.5507 | 45293.21 |
| Mean Imputation | RMSE | 607.5215 | 89.4746 | 1.9984 | 2.5763 | 1.1763 | 140.5494 |
|  | RMSLE | 0.0281 | 0.0357 | 0.1106 | 0.0368 | 0.0013 | 0.0425 |
|  | MAPE | 0.0065 | 0.0074 | 0.0308 | 0.0063 | 0.0002 | 0.0102 |
|  | PB | 0.0017 | -0.0015 | -0.0228 | -0.0024 | -0.0001 | -0.005 |
|  | MSE | 369082.4 | 8005.703 | 3.9936 | 6.6373 | 1.3836 | 75420.02 |
| Median Imputation | RMSE | 592.2845 | 89.4969 | 2.0221 | 2.7378 | 1.2359 | 137.5555 |
|  | RMSLE | 0.0275 | 0.0355 | 0.1119 | 0.0392 | 0.0013 | 0.0431 |
|  | MAPE | 0.0063 | 0.0073 | 0.0314 | 0.0066 | 0.0002 | 0.0104 |
|  | PB | 0.0009 | -0.0009 | -0.0237 | -0.0041 | -0.0001 | -0.0056 |
|  | MSE | 350801 | 8009.699 | 4.0887 | 7.4957 | 1.5276 | 71764.76 |
| Mode Imputation | RMSE | 693.9645 | 92.2426 | 3.0353 | 3.787 | 1.1808 | 158.842 |
|  | RMSLE | 0.0319 | 0.0361 | 0.1664 | 0.0512 | 0.0013 | 0.0574 |
|  | MAPE | 0.0075 | 0.0071 | 0.0282 | 0.0093 | 0.0002 | 0.0105 |
|  | PB | 0.0044 | 0.001 | 0.0238 | -0.009 | 0.0001 | 0.0041 |
|  | MSE | 481586.7 | 8508.7 | 9.213 | 14.341 | 1.3943 | 98024.08 |
| PMM Imputation | RMSE | 727.7675 | 85.1729 | 1.1414 | 1.6563 | 1.1058 | 163.3688 |
|  | RMSLE | 0.034 | 0.0339 | 0.0679 | 0.023 | 0.0012 | 0.032 |
|  | MAPE | 0.0078 | 0.0068 | 0.0139 | 0.0036 | 0.0002 | 0.0065 |
|  | PB | -0.0002 | -0.0006 | -0.0028 | -0.0003 | 0 | -0.0008 |
|  | MSE | 529645.5 | 7254.418 | 1.3028 | 2.7433 | 1.2229 | 107381 |
| Hotdeck Imputation | RMSE | 857.0318 | 127.4644 | 2.9139 | 3.6172 | 1.6325 | 198.5319 |
|  | RMSLE | 0.0407 | 0.0501 | 0.1451 | 0.0511 | 0.0018 | 0.0577 |
|  | MAPE | 0.0092 | 0.01 | 0.0393 | 0.0083 | 0.0003 | 0.0134 |
|  | PB | 0.0019 | -0.0014 | -0.0234 | -0.0026 | -0.0001 | -0.0051 |
|  | MSE | 734503.4 | 16247.16 | 8.491 | 13.0838 | 2.6651 | 150155 |

**RN:** Radon Concentration; **TH:** Thoron; **TC:** Temperature in degree Celsius; **RH:** Relative Humidity; **PR:** Pressure; **PMM:** Predictive mean matching

c)

| Imputation Methods | Performance  Measures | RN | TH | TC | RH | PMBR | Average |
| --- | --- | --- | --- | --- | --- | --- | --- |
| Proposed Methodology | RMSE | 571.4521 | 81.9031 | 0.9394 | 1.3211 | 0.8783 | 131.2988 |
|  | RMSLE | 0.0268 | 0.0318 | 0.0563 | 0.018 | 0.0009 | 0.0268 |
|  | MAPE | 0.0073 | 0.0076 | 0.0138 | 0.0034 | 0.0002 | 0.0065 |
|  | PB | 0.0001 | 0.0001 | -0.0025 | 0 | 0 | -0.0004 |
|  | MSE | 326557.5 | 6708.122 | 0.8825 | 1.7454 | 0.7713 | 66653.8 |
| Mean Imputation | RMSE | 710.9478 | 115.8893 | 2.4841 | 3.1497 | 1.3999 | 166.7742 |
|  | RMSLE | 0.0332 | 0.0457 | 0.1353 | 0.0446 | 0.0015 | 0.0521 |
|  | MAPE | 0.0092 | 0.0114 | 0.0457 | 0.0093 | 0.0003 | 0.0152 |
|  | PB | 0.002 | -0.002 | -0.0325 | -0.0031 | -0.0001 | -0.0071 |
|  | MSE | 505446.8 | 13430.32 | 6.1708 | 9.9207 | 1.9597 | 103779 |
| Median Imputation | RMSE | 695.3116 | 116.1786 | 2.5553 | 3.325 | 1.454 | 163.7649 |
|  | RMSLE | 0.0326 | 0.0456 | 0.1391 | 0.0473 | 0.0016 | 0.0532 |
|  | MAPE | 0.009 | 0.0113 | 0.0477 | 0.0097 | 0.0003 | 0.0156 |
|  | PB | 0.0008 | -0.0011 | -0.0362 | -0.0057 | -0.0001 | -0.0085 |
|  | MSE | 483458.3 | 13497.46 | 6.5297 | 11.0555 | 2.1142 | 99395.09 |
| Mode Imputation | RMSE | 775.5668 | 124.1154 | 3.3445 | 4.5859 | 1.4961 | 181.8217 |
|  | RMSLE | 0.0359 | 0.0479 | 0.1685 | 0.0618 | 0.0016 | 0.0631 |
|  | MAPE | 0.0101 | 0.0113 | 0.0364 | 0.0138 | 0.0004 | 0.0144 |
|  | PB | 0.0048 | 0.0033 | 0.0239 | -0.0133 | 0.0002 | 0.0038 |
|  | MSE | 601503.8 | 15404.64 | 11.1857 | 21.0308 | 2.2383 | 123388.6 |
| PMM Imputation | RMSE | 884.1799 | 156.9706 | 1.4022 | 2.0174 | 1.2741 | 209.1688 |
|  | RMSLE | 0.0414 | 0.0455 | 0.0847 | 0.0274 | 0.0014 | 0.0401 |
|  | MAPE | 0.0113 | 0.0107 | 0.0211 | 0.0053 | 0.0003 | 0.0097 |
|  | PB | -0.0013 | -0.0015 | -0.0053 | -0.0005 | 0 | -0.0017 |
|  | MSE | 781774.1 | 24639.78 | 1.9661 | 4.0698 | 1.6234 | 161284.3 |
| Hotdeck Imputation | RMSE | 1020.23 | 156.5929 | 3.5274 | 4.7114 | 1.9646 | 237.4052 |
|  | RMSLE | 0.0489 | 0.0617 | 0.1767 | 0.0664 | 0.0021 | 0.0712 |
|  | MAPE | 0.0131 | 0.0152 | 0.0564 | 0.0132 | 0.0005 | 0.0197 |
|  | PB | 0.0025 | -0.0014 | -0.0314 | -0.003 | -0.0001 | -0.0067 |
|  | MSE | 1040869 | 24521.35 | 12.4422 | 22.1972 | 3.8595 | 213085.8 |

**RN:** Radon Concentration; **TH:** Thoron; **TC:** Temperature in degree Celsius; **RH:** Relative Humidity; **PR:** Pressure; **PMM:** Predictive mean matching
