# Supplementary material for: Imputation by feature importance (IBFI): A methodology to envelop machine learning method for imputing missing patterns in time series data
Source: PLoS One. 2022 Jan 13;17(1):e0262131. doi: 10.1371/journal.pone.0262131 (PMC8758196; doi:10.1371/journal.pone.0262131)
Supplement: Attachment — Submitted filename: Response to Reviewers.docx [file pone.0262131.s005.docx]

Dear Editor and Editorial Staff,

PLOS ONE

We are pleased to inform you that we have revised the manuscript in light of reviewers’ comments. The anonymous reviewers’ recommendations were extremely useful and we have addressed all of their recommendations in the revised manuscript. Please see below for responses to each comment.

**Reviewer #1:**

| Comment | Please define SRGC in the introduction when it is mentioned for the first time. |
| --- | --- |
| Response | We have incorporated necessary changes in the introduction to address reviewers concern. |
| Comment | Response 2, lines 93-94: Please grammar-check |
| Response | We have proofread as well as grammatically checked the lines 93-94 in the revised manuscript. |
| Comment | Response 4: please grammar-check the description of various imputation methods that have been added to the introduction |
| Response | We have proofread as well as grammatically checked the description of various imputation methods in the revised manuscript. |
| Comment | Response 6: description of missingness mechanisms lacks clarity; please proofread |
| Response | To address the reviewer's concern, we have restructured and clarified the concept in a more precise and easy way. |
| Comment | Response 8: there is reference to PMM in the description of hot-deck imputation. This adds confusion since PMM is described in the next section. Further, the comment asked the authors to rephrase the description to remove jargon. However, the description is now too detailed which ultimately still falls short of explaining the method adequately (e.g, how is the donor pool picked?). An effort needs to be made to keep the description short by providing only the relevant information. |
| Response | Agreed with the findings of the respected reviewer. The reference in the hot-deck imputation section adds confusion when reading the description of the PMM imputation method. To address the concern, we have added relevant details of each imputation method and rephrased and proofread as well. |
| Comment | Response 9: this comment has not been addressed adequately. The mechanics of PMM have not been explained, nor have the parameters been described. |
| Response | To address this concern, we have explained the mechanics of PMM and its parameters in a more precise and easy way so that the concept can be easily figured out. |
| Comment | Response 10: The pseudo-code is still difficult to follow. |
| Response | To address the reviewer's concern, we have added comments and restructured some statements in the pseudo-code in a way that can be easily followed. |
| Comment | Response 14: The description of model reusability needs to be improved for readability, and then inserted in the manuscript. Also, while I agree that reusability reduces computation time, I am not sure how it results in more accurate predictions. Finally, I assume that “pure data” PD is used to create models. If so, that should be clarified. Presently, the purpose of PD has not been explained explicitly anywhere in the text. |
| Response | - The proposed methodology imputes the attributes or variables using a feature importance matrix. The sequence of imputation of values for the missing features in different samples differs from each other. The whole procedure is scanning the impure dataset as per the rejection threshold. Consider data row 1, the features $F1$ and $F5$ having a missing value while data row 100 have missing values at features $F1$, $F2,$and $F5$. Data row 1 has F2, F3, and F4 feature values available to predict the value of F1 and F5 whilst F3 and F4 have only available feature values to predict the missing value of F1, F2, and F5. During scan 1 and iteration 1, the feature importance matrix directs the algorithm to predict the missing value of F1 using the model fitted on features F2, F3, and F4 by considering F1 as a response and F2, F3, and F4 as predictor features. After predicting and imputing the missing value of F1, the model should store as M_1234_ for future patterns. Through the same scan when reached at data row 100, the feature importance matrix directs the algorithm to impute the value of F2 which is missing by taking F2 as a response attribute while F3 and F4 as predictor attributes. After predicting and imputing the missing value of F2, the data row 100 has now an available value of F2 to predict other features. Now consider the second iteration and scan 100, the feature importance matrix directs the algorithm to impute the value of F1 first instead of F5. Currently, F2, F3, and F4 are available values to predict the missing value of F1. Instead of fitting another model to predict the missing value of F1, the previously generated M_1234_ model is reused and values of F2, F3, and F4 are passed to that model to predict the missing value of F1. The reusability of the already fitted model to impute the similar missing patterns enhance the performance and reduces the time and space requirements. - The accuracy of predicting the missing value for different features is based upon the feature importance matrix and runtime decision of choosing the best available features for training while model reusability results in reducing the computation time. - Agreed with the respected reviewer. The pure data (PD) has all the feature values available to fit a machine learning model. To address this concern, we have explicitly explained the usage of PD in imputation by feature importance (IBFI). |
| Comment | Response 17: In Figure 7 of the revision, what is the measurement number? You mentioned "scans" and "iterations" in response 14. How does it relate to that? |
| Response | - In IBFI, “scan” traverses the whole impure data by scanning each data row to check for missing patterns and selection of best available features for training. On the other hand, “iteration” consists of one full scan of the impure data. During each iteration, the number of missing features per data row gets reduced by one feature. - In figure 7, the measurement number represents the data rows handled for imputing missing patterns by IBFI during the subsequent scans and iterations. |
| Comment | Response 19: Please state your assumption justifying the use of a rejection threshold of 3 in the manuscript. |
| Response | If there is an increasing number of missing values in different data rows, there is the chance that imputing the values may result in contamination of those data rows and result in poor analyses or biased results on further experimentation using that imputed dataset.   - The dataset used in this study has 5 features. The assumption behind the use of a rejection threshold of 3 is to ensure that at least 2 features are available for model training. The imputation of more than 3 out of 5 features may result in a relatively less accurate imputation of missing value because of the use of previously imputed value for the prediction of the other missing feature values. |
| Comment | It seems that PLOS data policy requires data underlying the findings to be fully available, which includes the data points behind the summary statistics. I only see the summary statistics in the supplement. |
| Response | The data that support the findings of this study are available from the corresponding author upon reasonable request. |

Sincerely,

Prof. Dr. M. Rafique
